# Tumor-stroma ratio combined with PD-L1 identifies pancreatic ductal adenocarcinoma patients at risk for lymph node metastases

**DOI:** 10.1038/s41416-025-03019-z

**Published:** 2025-04-18

**Authors:** Xianlong Chen, Shanyue Sun, Jiapeng Zhao, Shuangni Yu, Jie Chen, Xinyuan Chen

**Affiliations:** 1 Department of Pathology, Peking Union Medical College Hospital, Chinese Academy of Medical Sciences & Peking Union Medical College, Beijing, China; 2 Shandong Provincial Hospital Affiliated to Shandong First Medical University, Jinan, China; 3 4+4 Medical Doctor Program, Chinese Academy of Medical Sciences & Peking Union Medical College, Beijing, China

**Keywords:** Pancreatic cancer, Prognostic markers

## Abstract

**Background:**

Pathological examination of lymph node metastasis (LNM) is crucial for treating pancreatic ductal adenocarcinoma (PDAC). Although the tumour stroma is correlated with prognosis in multiple solid tumors, its role in detecting LNM in PDAC is unclear. Thus, this study aimed to investigate the relationship of tumor-stroma ratio (TSR) with LNM, survival and mutational profile in PDAC.

**Methods:**

In this multicenter retrospective study, we examined molecular and clinicopathologic features of 737 PDAC patients from 5 independent cohorts, including surgically resected and endoscopic ultrasound fine-needle aspiration (EUS-FNA) biopsy specimens. TSR was evaluated on hematoxylin and eosin-stained slides and classified as stroma-low (<50% stroma) or stroma-high (≥50% stroma).

**Results:**

Compared to TSR-high cases, TSR-low cases were significantly associated with LNM (*P* < 0.001). TSR could accurately distinguish patients with and without LNM with an area under curve (AUC) of 0.749, with the sensitivity and specificity of 76.5% and 71.6%, respectively. This accuracy of TSR for identifying LNM was further increased by adding other factors including PD-L1 expression or pretreatment serum CA19-9 levels. TSR showed similar levels of accuracy in analysis of resected tumor specimens and EUS-FNA biopsies. Moreover, we found that TSR could also identify residual nodal involvement after neoadjuvant therapy (NAT) using pretreatment EUS-FNA biopsy samples. Heterogeneous genetic alterations were observed between TSR-low and TSR-high subgroups. TSR was identified as an independent predictor of LNM and worse disease-free survival. Major findings were all reproducible in validation, EUS-FNA biopsy, and pre-treatment NAT EUS-FNA biopsy cohorts.

**Conclusions:**

TSR served as a robust and reproducible biomarker that identifies patients at risk for LNM. TSR might be used to select treatment and management strategies for PDAC patients.

## Background

Pancreatic ductal adenocarcinoma (PDAC), accounting for more than 90% of all pancreatic malignancies [1], is the fourth leading cause of cancer-related deaths in developed countries with a 5-year survival rate of less than 8% [2–4]. The morbidity rates of PDAC in both sexes have increased in recent decades. In the past few years, the survival of patients with PDAC has made little improvement, because of late diagnosis and resistance to various therapies [5, 6]. Less than 20% of patients can be treated surgically [2, 7]. Even for patients who undergo surgical treatment, the 5-year survival rate is lower than 20%, and 80% of these patients experience recurrence within 2 years [8]. Multidisciplinary therapeutic approaches for more effective PDAC treatment, including neoadjuvant therapy (NAT), are extensively investigated [9, 10]. The National Comprehensive Cancer Network (NCCN) guidelines recommend NAT for borderline resectable cancers and certain high-risk subsets of patients with resectable cancers, including those with lymph node involvement, high carbohydrate antigen 19-9 (CA19-9) levels, and large tumor sizes.

PDAC tends to metastasize to regional lymph nodes, increasing the possibility of distant metastasis and substantially poorer prognosis [11, 12]. Patients with PDAC and lymph node metastasis (LNM) can benefit from NAT followed by surgery, prolonging survival and increasing the time to relapse [13]. However, most patients with PDAC do not respond to NAT and are at risk of missing the optimal window for curative surgery [14, 15]. Thus, accurate LNM diagnosis is critical for clinical decision-making and patient counseling, but preoperative LNM assessment in patients with PDAC is challenging. Clinically, the nodal status is evaluated using endoscopic ultrasound (EUS), positron emission tomography, magnetic resonance imaging, or computed tomography with relatively inadequate sensitivity and specificity, contributing to a high rate of missed LNM diagnoses [16–18]. To overcome the challenges of imaging-based diagnostic strategies, histopathologic or molecular biomarkers should be integrated, but little research has been conducted.

Growing evidence suggests that tumor invasion and metastasis not only depend on the biological behavior of cancer cells, but that the interaction between cancer cells and their stroma also plays an important role. More attention has been paid to tumor stroma, which is composed of extracellular matrix, fibroblasts, immune cells, and endothelial cells. The stroma plays an important role in tumor growth, invasion, progression, metastasis, and resistance to treatment [19–23]. Thus, the tumor stroma holds many possibilities in the search for markers to predict the prognosis and effect of treatment. The tumor-stroma ratio (TSR) reflects the proportion of cancer-related stroma relative to cancer cells. The TSR scoring, which is determined on hematoxylin and eosin (H&E)-stained slides, which do not incur any additional costs, is a fairly simple and quick process that can be incorporated into daily pathological diagnosis. Furthermore, evaluation of the TSR has shown good inter-observer agreement [24–26]. Recently, the TSR has been identified as a promising prognostic factor in various types of solid malignancies, including ovarian cancer, triple-negative breast cancer, and FIGO stage IIIC cervical carcinoma [27–29]. However, the clinical significance as TSR to serve as a biomarker to identify LNM pre-operatively in PDAC, through a systematic and comprehensive analysis in large, multiple independent patient cohorts has not been attempted. Availability of such a biomarker will facilitate physicians in making more informed clinical decision-making and developing individualized treatment strategies for improved management of PDAC patients.

The main goal of this study was to evaluate if TSR could identify PDAC patients at risk for lymph node metastases. Additionally, a comprehensive biomarker evaluation based on TSR combining other pathological features for LNM detection in PDAC was conducted. The diagnostic performance was validated using two large-scale independent clinical cohorts. To further translate our findings into a clinically viable scenario, we confirmed the robustness of our approach using pre-treatment EUS-FNA biopsy specimens. We also examined the associations of TSR with mutational profile and survival outcome.

## Methods

### Patient cohorts

We retrospectively recruited four independent large-scale cohorts of PDAC patients with corresponding specimens across two institutions. A training cohort (*N* = 105) was recruited at the Peking Union Medical College Hospital from 2009 to 2022. We also enrolled a validation cohort (*N* = 272) at both Peking Union Medical College Hospital and Shandong Provincial Hospital from 2012 to 2021 to further validation. We then enrolled another independent retrospective PDAC cohort with EUS-FNA biopsy specimens (*N* = 75) to evaluate the robustness using EUS-FNA biopsy specimens. To further assess the performance of TSR in predicting LNM, we collected 103 EUS-FNA biopsy specimens from patients with PDAC who underwent NAT with surgery. EUS-FNA biopsy specimens were collected before treatment initiation and processed in accordance with standard diagnostic procedures using endoscopic and cytological methods. All slides from patients were retrieved, scrutinized, and reclassified by 2 experienced pancreatic pathologists. Tumors were staged using the American Joint Committee on Cancer (AJCC) system (version 8), and LNM status was assessed by histopathological examination of the resected lymph nodes. Based on clinical guidelines, the cutoff value for CA19-9 was set at 37 U/ml [30]. The clinicopathologic and follow-up data were extracted from electronic medical records. This study was approved by the Ethics Review Committees of Peking Union Medical College Hospital (S-K1593) and Shandong Provincial Hospital (No.2022 − 178). All participants signed the informed consent form that had also been approved by the Ethics Committees. All procedures involving human tissues were performed in accordance with the principles of the 1964 Declaration of Helsinki and related declarations.

### TCGA cohort

The hematoxylin and eosin (H&E)-stained slides, somatic mutation, genome-wide mRNA data, and corresponding clinicopathologic information were downloaded from The Cancer Genome Atlas-Pancreatic Adenocarcinoma (TCGA-PAAD) archives, including 182 cases.

### Assessment of the TSR

For each tumor sample, two H&E-stained slides were selected for TSR scoring. The most invasive tumor areas were chosen from the whole slide under a 4× microscope objective. Then, a 10× objective was used to identify fields in which both tumor and stromal components were contained. Tumor cells were observed in all borders within each examined field. If stromal features were observed in different fields and sections of a single patient, we selected the area with the highest proportion of stroma for the TSR scoring of that patient.

**Fig. 1:**
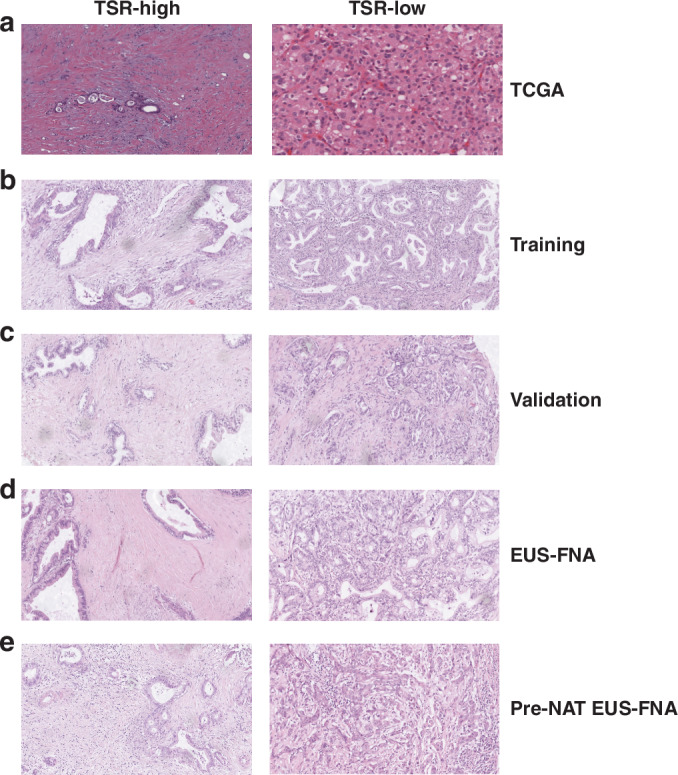
Representative images of TSR-high and -low specimens. **a**–**e** The representative images in the TCGA (**a**), training (**b**), validation (**c**), EUS-FNA (**d**), and pre-NAT EUS FNA (**e**) cohorts (original magnification, ×200).

TSR was evaluated by two independent investigators (XL. C. and XY. C.) who were blinded to each other’s scores. In cases of discrepancy, a third investigator (SN. Y.) made the final decision. A TRS ≥ 50% was classified as high-stromal, while TRS < 50% was considered as low-stromal, as described by Mesker et al. [28]. Representative images of high-stromal and low-stromal cases are presented in Fig. 1a–e.

### Somatic mutation analysis

TCGA-PAAD somatic mutation datasets were gathered in a MAF-like format, achieved in excel and compiled to a CSV file. The somatic mutation data was annotated by Genome Reference Consortium human genome build 38 (hg38). Then R package ‘maftools’ was utilized to produce the oncoplot in two subgroups, together with co-occurrence of mutations. Co-occurrence and mutual exclusivity of DNA somatic mutation refer to two or more genes that prone to be positively or negatively correlated in DNA mutation among different groups.

### Immunohistochemical assessment of PD-L1 expression

Immunohistochemical staining was performed using an automated immunostainer (BOND-III, Leica Biosystems, Wetzlar, Germany) with primary antibodies against PD-L1 from Cell Signaling Technology (13684S). Positive PD-L1 expression on tumor cells was based on the tumor proportion score (TPS), calculated as the number of PD-L1-expressing tumor cells divided by the total number of viable tumor cells. Based on clinical practice and previous studies, the TPS cutoff was set at 1% [31, 32].

### Statistical analysis

Continuous variables are presented as median (range), and categorical variables are described as frequency (percentage). The Pearson’s χ^2^ test or Fisher’s exact test was applied as appropriate to assess the associations between categorical variables. Spearman correlation coefficients were used to analyze the relationships between continuous variables. Kaplan-Meier curves with log-rank test were plotted to assess DSS, following multivariate Cox analysis using the significant variables which associated with survivals (*P* < 0.05). Univariate and multivariate logistic regression analyses were used to estimate the odds ratios (ORs) for LNM, of which the predictive accuracy was quantified using the area under the receiver operating characteristic (ROC) curve (AUC). All statistical tests were analyzed in R version 4.3.3. Statistical significance was defined as a two-sided *P*-value < 0.05.

## Results

### Association of TSR with clinicopathologic characteristics

To explore the clinical significance of TSR, we first assessed TSR in three large-scale independent clinical cohorts without NAT (Fig. 1). We analyzed the distribution of TSR in PDAC by subgrouping based on differentiation, perineural invasion, lymphovascular invasion (LVI), T stage, N stage, and AJCC stage. The associations of TSR with the clinicopathologic parameters are summarized in Supplemental Table S1. In this study, 51.4%, 40.1%, and 52.0% of the tumor specimens were identified as TSR-high in the training, validation, and EUS-FNA biopsy cohorts, respectively. In the training cohort, low TSR was significant associated with LNM (*P* < 0.001) and advanced AJCC stage (*P* = 0.005; Supplemental Table S1). Consistent with the training cohort, TSR was found to be correlated with LNM (validation cohort: *P* < 0.001; EUS-FNA cohort: *P* = 0.003) and AJCC stage (validation cohort: *P* < 0.001; EUS-FNA cohort: *P* = 0.001; Supplemental Table S1) as well in both the validation and EUS-FNA cohort. In summary, TSR was related to aggressive clinical phenotypes in PDAC.

### Association of TSR with molecular characteristics

To determine the effects of TSR on the molecular features of PDAC, we explored the somatic mutation profiles from the TCGA cohort. Missense variations were the most common mutation type in the both TSR-high and TSR-low subgroups. we found mutations of the *KRAS* and *TP53* gene were most frequent in both subgroups with the mutation rates higher than 40%. Noteworthily, *SMAD4* mutations were more common in the TSR-high subgroup with the mutation rate of 20%, whereas *CDKN2A* mutations were more common in the TSR-low subgroups (Supplemental Figure S1A, B). Additionally, we explored the gene interactions in the top 20 genes with the highest mutation rates. As a result, we found *CDKN2A* mutations were significantly co-occurrent with *KRAS* mutations in the TSR-low subgroups, while mutations of *SMAD4* genes were significantly co-occurrent with *KRAS* mutation in the TSR-high subgroup (Supplemental Figure S1C, D). Therefore, TSR could identify distinct mutation patterns in PDAC.

### The diagnostic performance of TSR for detecting LNM in patients with PDAC

**Fig. 2:**
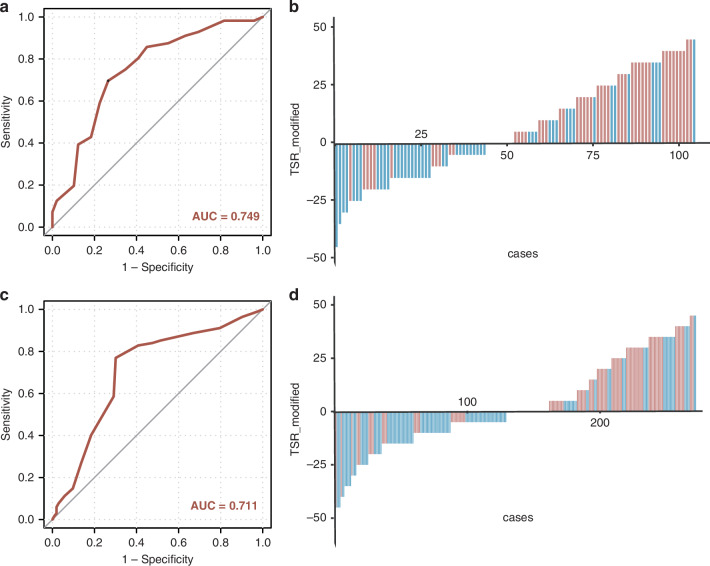
Training and Validation of TSR for the Detection of LNM in Surgically Resected Specimens from Patients with PDAC. **a**, **c** ROC curves of TSR for predicting LNM in the training (**a**) and validation (**c**) cohorts. **b**, **d** Modified TSR distribution plot in the training (**b**) and validation (**d**) cohorts. LNM lymph node metastasis, TSR tumor-stroma ratio, PDAC pancreatic ductal adenocarcinoma, ROC receiver operating characteristic.

The LNM status significantly alters the survival, treatment outcomes of patients with PDAC, and identification of high-risk subgroups. Among our three independent cohorts, the TSR was observed to be significantly associated with LNM status. Thus, we hypothesize that the TSR could effectively help to identify patients with PDAC at the risk of LNM. To confirm our hypothesis, we examined the diagnostic robustness of TSR in the clinical cohorts. In the training cohort, 18 of 54 TSR-high cases had positive lymph nodes, while 38 of 51 TSR-low cases had positive lymph nodes (*P* < 0.001). TSR was highly robust when detecting LNM in patients with PDAC (AUC = 0.749; Fig. 2a, b) with the sensitivity and specificity of 76.5% and 71.6%, respectively. Positive predictive value (PPV) was 67.9%, and negative predictive value (NPV) was 75.0%. Logistic regression analysis comparing cases with LNM to those without is shown in Table 1. Univariate analysis showed that TSR, CA19-9, and PD-L1 expression were significantly associated with LNM. Multivariate logistic analysis further confirmed that TSR was an independent predictive factor of LNM in patients with PDAC (OR = 6.90, 95% CI = 2.79–17.08, *P* < 0.001; Table 1).

**Table 1:** Predictors of lymph nodes metastasis on univariable logistic analysis in the training, validation, EUS-FNA and pre-NAT EUS-FNA cohort.

Variables	Training Cohort						Validation Cohort						EUS-FNA Cohort						pre-NAT EUS-FNA Cohort					
	Univariate			Multivariate			Univariate			Multivariate			Univariate			Multivariate			Univariate			Multivariate		
	OR	95%CI	*P*	OR	95%CI	*P*	OR	95%CI	*P*	OR	95%CI	*P*	OR	95%CI	*P*	OR	95%CI	*P*	OR	95%CI	*P*	OR	95%CI	*P*
**Age**(>60 yrs)	0.77	0.35–1.70	0.523				0.66	0.40–1.09	0.106				0.36	0.14–0.93	**0.034**	0.55	0.19–1.60	0.272	0.71	0.24–2.10	0.537			
**Sex**(Male)	1.18	0.55–2.55	0.675				1.04	0.64–1.70	0.867				1.18	0.48–2.94	0.720				1.87	0.60–5.84	0.280			
**Differentiation**(Poor)	0.89	0.40–2.01	0.782				1.53	0.91–2.57	0.107				NA						NA					
**PNI**(Yes)	0.56	0.24–1.28	0.169				1.26	0.75–2.12	0.380				NA						NA					
**LVI**(Yes)	1.39	0.63–3.04	0.412				1.68	1.00–2.83	**0.050**	1.58	0.87–2.87	0.130	NA						NA					
**CA19-9**( ≥ 37)	2.10	1.21–3.79	**0.041**				2.07	1.15–3.72	**0.015**	1.79	0.91–3.53	0.093	1.79	1.32–5.17	**0.031**				2.67	1.56–12.62	**0.043**			
**Location**(Head)	1.76	0.81–3.82	0.152				1.90	1.15–3.14	**0.012**	1.37	0.77–2.46	0.285	4.90	1.67–14.34	**0.004**	4.21	1.28–13.84	**0.018**	1.29	0.43–3.87	0.647			
**pT Stage**(T3-T4)	1.58	0.67–3.72	0.292				0.99	0.57–1.73	0.974				0.95	0.33–2.77	0.931				0.16	0.05–0.53	**0.003**	0.02	0.00–0.20	**0.001**
**AJCC** (III–IV)	17.56	2.22–138.71	**0.007**				11.31	3.95–32.35	**<0.001**				Inf	0.00–Inf	0.992				Inf	0.00–Inf	0.993			
**TSR**(Low)	5.85	2.51–13.63	**<0.001**	6.90	2.79–17.08	**<0.001**	7.65	4.40–13.28	**<0.001**	7.38	4.15–13.12	**<0.001**	4.16	1.57–11.01	**0.004**	4.24	1.40–12.82	**0.010**	3.01	1.00–9.07	**0.050**	20.91	2.03–214.85	**0.010**
**PD-L1(TPS)**(Positive)	2.67	1.16–6.17	**0.021**	3.50	1.35–9.04	**0.010**	2.23	1.28–3.89	**0.005**	2.44	1.30–4.59	**0.006**	2.89	1.03–8.10	**0.044**	3.07	0.94–10.00	0.062	7.87	2.44–25.45	**<0.001**	7.56	1.77–32.32	**0.006**
**TSR** + **PD-L1(TPS)**																								
High/Negative	Reference						Reference						Reference						Reference					
High/Positive	3.25	1.00–10.59	**0.048**				4.07	1.72–9.66	**0.001**				3.33	0.72–15.54	0.125				10.44	1.66–65.82	**0.012**			
Low/Negative	6.50	2.22–19.01	**<0.001**				10.96	5.47–21.94	**<0.001**				4.62	1.35–15.78	**0.015**				3.36	0.52–21.60	0.202			
Low/Postive	26.00	4.89–138.11	**<0.001**				15.67	6.48–37.85	**<0.001**				18.00	2.92–110.96	**0.002**				16.45	2.97–91.23	**0.001**			

*PNI* perineural invasion, *LVI* lymphovascular invasion, *TSR* tumor-stroma ratio, *PD-L1* Programmed cell death 1 ligand 1, *AJCC* The American Joint Committee on Cancer, *OR* odds ratio, *CI* confidence interval.

*p*-values < 0.05 are bolded.

Next, we validated our findings in an independent validation cohort from another hospital. Consistent with previous findings, TSR exhibited higher predictive accuracy (AUC = 0.711) with a higher sensitivity (85.7%) and tolerable specificity (63.3%), and was an independent risk factor (OR = 7.38, 95% CI = 4.15–13.12, *P* < 0.001; Fig. 2c, d; Table 1) for detecting LNM in PDAC. PPV and NPV were 72.7% and 79.5%, respectively.

### High-order validation of TSR for detecting LNM in the EUS-FNA biopsy cohort

**Fig. 3:**
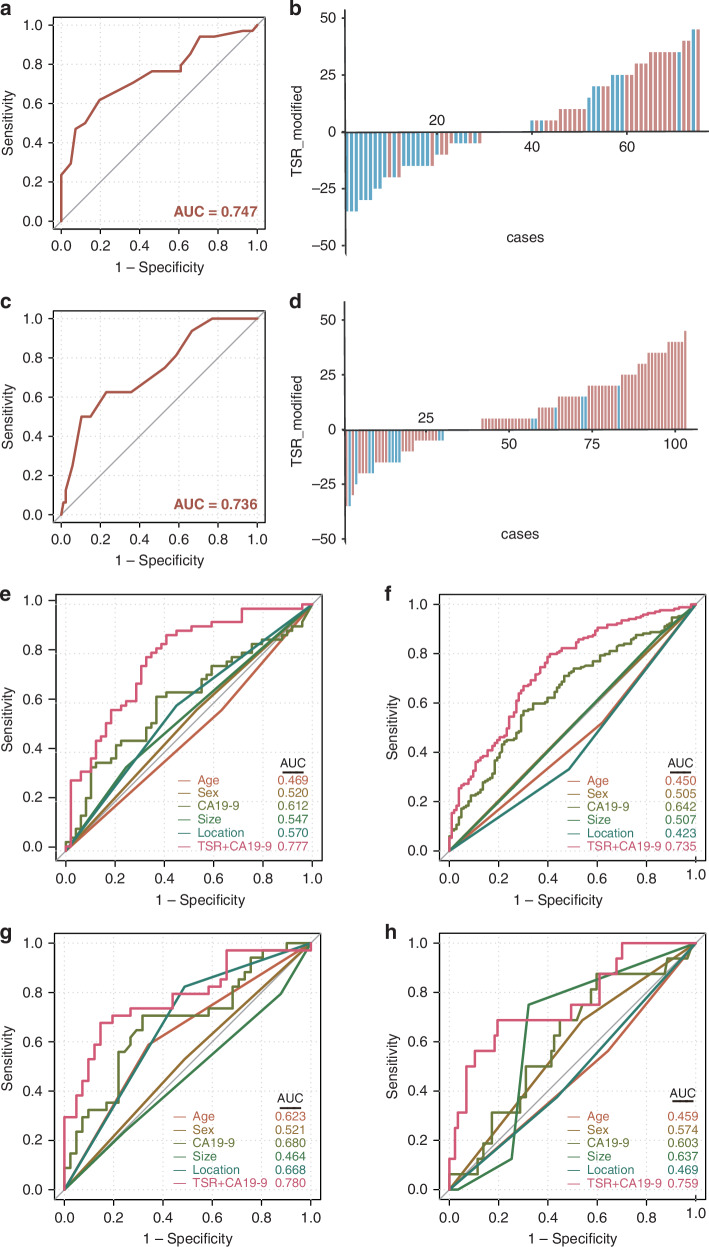
Training and validation of TSR for the detection of LNM in EUS-FNA biopsy specimens and combination of clinicopathologic characteristics from patients with PDAC. **a,** **c** ROC curves of TSR for distinguishing LNM in the EUS-FNA (**a**) and pre-NAT EUS-FNA (**c**) cohorts. **b**, **d** Distribution plot of modified TSR in the EUS-FNA (**b**) and pre-NAT EUS-FNA (**d**) cohorts. **e**–**h** ROC curves of the TSR combining with clinicopathologic characteristics in the training (**e**), validation (**f**), EUS-FNA (**g**) and pre-NAT EUS-FNA (**h**) cohorts. EUS-FNA indicates endoscopic ultrasound fine-needle aspiration, LNM lymph node metastasis, NAT neoadjuvant therapy, TSR tumor-stroma ratio, PDAC pancreatic ductal adenocarcinoma, ROC receiver operating characteristic.

While the validation of TSR using surgically resected tissue specimens was necessary for LNM detection, validation of TSR in pre-treatment biopsy specimens would pave a path for an easier translation of TSR in clinical settings. Physicians would then be able to categorize high-risk patients with otherwise unresectable tumors and give them more informed recommendations for NAT. Therefore, we enrolled 75 PDAC patients with EUS-FNA biopsy specimens deemed resectable following the NCCN guidelines. Consistent with the training and validation cohort, TSR was identified as an independent risk factor with a satisfactory AUC of TSR for detecting LNM in the EUS-FNA cohort (AUC = 0.747; Fig. 3a, b; OR = 4.24, 95% CI = 1.40–12.82, *P* = 0.010; Table 1). Comparable sensitivity, specificity, PPV, and NPV (80.5%, 61.8%, 71.7%, 72.4%, respectively) were displayed in the EUS-FNA biopsy cohort than the training and validation cohorts.

### Performance of TSR for predicting residual nodal involvement following NAT in EUS-FNA biopsy samples

Multidisciplinary strategies, including NAT, play an increasing pivotal role in the treatment of patients with resectable and borderline resectable PDAC. The residual lymph nodal status, modified by NAT (ypN), is a well-established prognostic factor in PDAC. We assumed that TSR could predict the ypN status in pre-treatment EUS-FNA biopsy cohort. Based on the hypothesis, we enrolled 103 patients who undergoing NAT followed by surgery with pre-treatment EUS-FNA biopsy specimens. The clinicopathologic characteristics were illustrated in Supplemental Table S2, showing significant association between TSR and ypN (*P* = 0.044). In this cohort, the satisfactory accuracy of TSR for detecting ypN was displayed with an AUC of 0.736 (Fig. 3c, d). The sensitivity was 80.5%, and the specificity was 59.2%. PPV and NPV were 90.7% and 47.1%, respectively. In addition, TSR was identified as an independent factor of LNM using univariate and multivariate analyses (OR = 20.91, 95% CI = 2.03–214.85, *P* = 0.010; Table 1).

### Higher accuracy for LNM detection by combining TSR with serum CA19-9 levels

CA19-9 is a well-established serum biomarker for diagnosis and management of PDAC. Hence, we hypothesized that increased accuracy of detecting LNM could be observed when combining TSR with serum CA19-9 levels. Consistent with our hypothesis, the combination models exhibited improved predictive accuracy in both training and validation cohorts (the training cohort: AUC = 0.777; the validation cohort: AUC = 0.735), outperforming other clinicopathologic characteristics, including age, sex, sole serum CA19-9 levels, tumor size, and location (Fig. 3e, f). Moreover, the combining models showed increased sensitivity (Training: 87.5%; Validation: 79.9%) and tolerable reduction of specificity (Training: 59.2% Validation: 59.2%) than sole TSR for predicting LNM in both cohorts. The PPV and NPV were 71.0% and 80.6%, respectively in the training cohort, and 76.3% and 64.2%, respectively in the validation cohort. In agreement with these findings, we also demonstrated elevated accuracy of combination models when detecting LNM in the EUS-FNA biopsy cohort (AUC = 0.780, sensitivity: 67.6%, specificity: 85.4%; Fig. 3g). Consistent with the EUS-FNA cohort, the combination of TSR with serum CA19-9 levels enhanced the diagnostic accuracy of ypN status in the pre-NAT EUS-FNA biopsy cohort (AUC = 0.759; Fig. 3h), highlighting the diagnostic potential of the model combining TSR with serum CA19-9 levels.

### Higher accuracy for LNM detection by combining TSR with PD-L1 expression

**Fig. 4:**
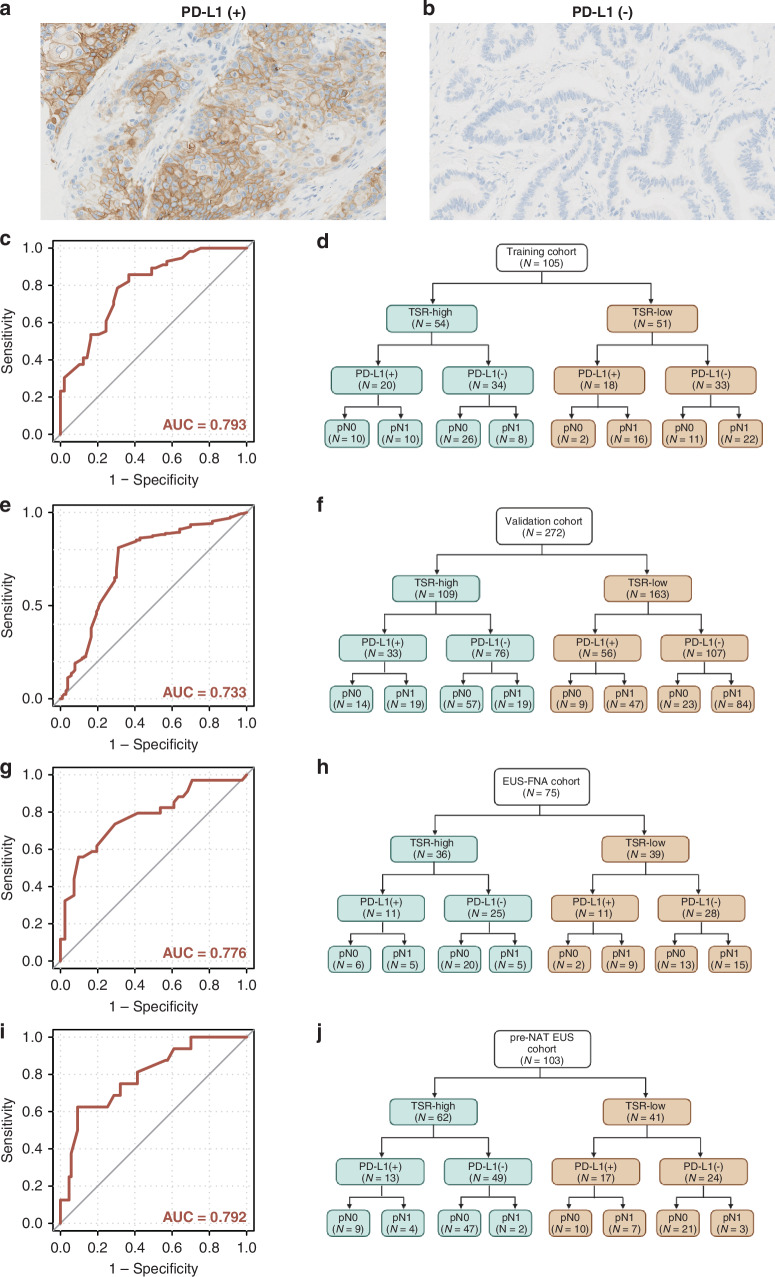
Predictive performance of combination of TSR and the protein expression of PD-L1 for detecting of LNM in patients with PDAC. **a**, **b** Representative IHC images of PD-L1-positive and -negative specimens (original magnification, ×400); **c**, **e**, **g**, **i** ROC curves of the models combining TSR and PD-L1 protein expression for detecting LNM status in the training (**c**), validation (**e**), EUS-FNA (**g**) and pre-NAT EUS-FNA (**I**) cohorts. **d**, **f**, **h**, **j** Pattern-based interpretation of TSR and PD-L1 positivity in the classification of PDAC with association to lymph node Metastasis in the training (**d**), validation (**f**), EUS-FNA (**h**) and pre-NAT EUS-FNA (**j**) cohorts. EUS-FNA endoscopic ultrasound fine-needle aspiration, PD-L1 programmed cell death 1 ligand 1, TSR tumor-stroma ratio, PDAC pancreatic ductal adenocarcinoma.

Based on the previous findings that PD-L1 promoter methylation and expression could predict LNM status accurately in patients with PDAC, we opted to explore whether integrate the TSR and PD-L1 TPS could improve the diagnostic robustness for detecting LNM. Of the 54 cases with TSR-high in the training cohort, we identified 34 cases with PD-L1 negative and 20 cases with PD-L1 positive (Fig. 4a, b). The remaining 51 TSR-low cases demonstrated a PD-L1 negative (*N* = 33) and PD-L1 positive (*N* = 18) pattern as well. LNM were presented in 16 of 18 cases with PD-L1 positive and TSR low, but only 8 of 26 cases with PD-L1 negative and TSR high. The satisfactory AUC of combination of TSR and PD-L1 positivity was demonstrated in the training cohort (AUC = 0.793; Fig. 4c, d). Increased sensitivity (85.7%) and minimum reduction of specificity (63.3%) were exhibited for LNM detection. The PPV and NPV were 72.7% and 79.5%, respectively. The robustness was validated in the validation cohort with elevated sensitivity (81.1%) and minimum reduction of specificity (68.9%) as well (AUC = 0.733; Fig. 4e, f). Furthermore, elevated accuracy using the combination models was also observed in the EUS-FNA biopsy cohort (AUC = 0.776; sensitivity 55.9%, specificity: 90.2%; Fig. 4g, h), further confirming the clinical significance of TSR combined with PD-L1 expression in evaluating the LNM in preoperative settings. Additionally, consistent with the three cohorts mentioned above, the combination of TSR with PD-L1 TPS enhanced the diagnostic accuracy of ypN status in the pre-NAT EUS-FNA biopsy cohort (AUC = 0.792; Fig. 4i, j), which may benefit physicians to make optimized treatment choices.

### Prognostic potential of TSR for patients with PDAC

**Fig. 5:**
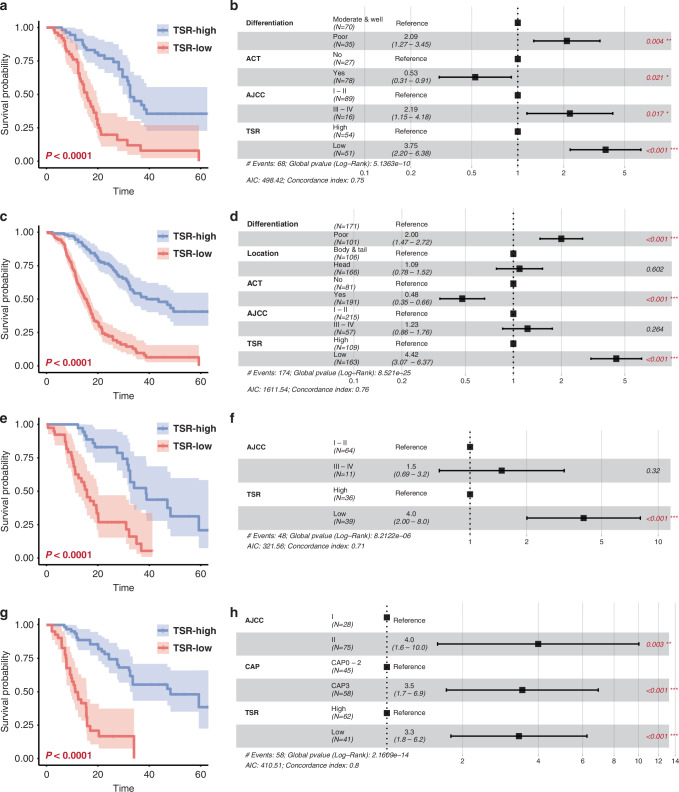
Prognostic Significance of TSR in Three Independent PDAC Cohorts. **a**, **c**, **e**, **g** Kaplan-Meier curves for disease-specific survival (DSS) by TSR in the training (**a**), validation (**c**). EUS-FNA (**e**), and pre-NAT EUS-FNA (**g**) cohorts. **b**, **d**, **f**, **h** Multivariate Cox analysis for DSS in the training (**b**), validation (**d**). EUS-FNA (**f**), and pre-NAT EUS-FNA (**H**) cohorts. *P*-values < 0.05 are marked in red. EUS-FNA indicates endoscopic ultrasound fine-needle aspiration; DSS disease-specific survival; TSR tumor-stroma ratio, PDAC pancreatic ductal adenocarcinoma.

Next, we compared the survival outcomes by TSR status in patients with PDAC. In the training cohort, Kaplan-Meier curves revealed that patients with high TSR possessed a higher probability of DSS at all-time points during follow-up (*P* < 0.001; Fig. 5a). Multivariate Cox analysis further demonstrated that the TSR status was an independent predictor of DSS in the training cohort (HR = 3.75, 95% CI = 2.20–6.38, *P* < 0.001; Fig. 5b and Supplemental Table S3). In line with these results, low TSR was significantly associated with poor survival outcomes, independent of other clinicopathologic characteristics in the validation cohort (HR = 4.42, 95% CI = 3.07–6.37, *P* < 0.001; Fig. 5c, d, and Supplemental Table S3). Higher-order validation from the results of Kaplan-Meier and multivariate Cox analyses in the EUS-FNA biopsy cohort confirmed the association of TPS with prognosis in the patients with PDAC (HR = 4.00, 95% CI = 2.00–8.00, *P* < 0.001; Fig. 5e, f, and Supplemental Table S3). As for patients undergoing NAT, lower TSR was also significantly associated with worse DSS (*P* < 0.001; Fig. 5g), and was an independent risk factor regarding other clinicopathologic variables in the pre-NAT EUS-FNA biopsy cohort (HR = 3.30, 95% CI = 1.80–6.20, *P* < 0.001; Fig. 5h and Supplemental Table S4), highlighting the satisfactory prognostic value of TSR in patients with PDAC regarding the therapy selections.

## Discussion

To systematically uncover the clinical significance of TSR in PDAC, we recruited four independent large-scale retrospective multi-center cohorts and demonstrated the subgroups categorized by TSR status exhibited distinct clinicopathologic characteristics, molecular alterations, immune cell infiltration, and survival outcomes. Subsequently, we developed and validated a robust diagnostic model based on TSR to detect LNM in patients with PDAC using both surgically resected and EUS-FNA biopsy specimens, which could be enhanced by combining with PD-L1 TPS and serum CA19-9 levels.

In the context of PDAC, the LNM status has been identified as a well-established risk factor to vastly influence survival and therapy selection, but preoperative LNM detection remains challenging [33]. Limited studies have focused on this topic, few of which were validated using EUS-FNA biopsy specimens. In our study, we provided a robust method based on TSR, a clinical assessable property of the TME, to accurately detect LNM status in patients with PDAC. Also, this model exhibited comparable accuracy in surgically resected and EUS-FNA biopsy specimens. Several previous studies have established models to diagnose LNM in patients with PDAC, some of which also highlighted the application of EUS-FNA biopsy specimens. Compared to these current models [34–36], our model showed superior or comparable accuracy. Besides, since the TSR was a readily, assessable, and high-reproducible index based on H&E staining slides, our model was more clinically viable and technically robust than other models based on mRNA or miRNA. These findings highlighted the diagnostic significance of our model for detecting LNM status in patients with PDAC.

Multidisciplinary therapy, including NAT, plays a crucial role in the individualized treatment of PDAC patients [37, 38]. the NCCN guidelines recommended the application of NAT in cases of resectable PDAC presenting with high-risk factors, including LNM. Therefore, providing superior risk stratification than other clinicopathologic characteristics, the development of robust biomarkers capable of determining LNM using pre-treatment EUS-FNA biopsy specimens is pivotal in clinical practice [36]. Our model, validated in pretreatment EUS-FNA biopsy samples, demonstrated the satisfactory accuracy in predicting the ypN status, further elevated by combining PD-L1 TPS and serum CA19-9 levels. These findings highlight the clinical significance of our model in pretreatment EUS-FNA specimens for individualized treatment, particularly NAT, in patients with PDAC. Accurate and cost-effective predictive biomarkers of treatment response are needed to identify patients most likely to benefit from standard treatments, and TSR can easily be implemented and incorporated into future prospective clinical trial designs and help to identify patients who are least likely to benefit long-term from NAT at the initial time of diagnosis.

Importantly, few previous studies investigated the prognostic value of the TSR by considering other clinically important parameters in patients with PDAC, and no unified conclusions were drawn. Three recent studies concluded that PDAC patients with a low-TSR status had a shorter overall survival [39–41], similar to our results. These studies focused only on overall survival, while progression is also an important issue for pancreatic cancer. Although Bo et al. explored the prognostic value in different TNM stages based on the Kaplan‒Meier method [41], multivariate analyses were not applied to assess the interaction between the TSR and TNM stage. The effect of the TSR stratified by other variables has not been described previously. Moreover, the biological functions of stroma are debating in PDAC. A previous study has revealed that the stroma can restrain tumor growth via decreasing vascularity and reducing tumor proliferation in PDAC [42]. However, Samara P Singh et al. found that the tumor stroma, containing abundant protumorigenic inflammatory cancer-associated fibroblasts, induces malignant transformation and chemotherapy resistance of PDAC [43]. Additionally, Zeyin Rong et al. revealed that dense stroma was associated with high TGF-β1/FBW7 ratio and could predict unfavorable prognoses in glycolytic subtype of PDAC [44]. Interestingly, in this study, we found that the patients with low TSR possessed higher mutation rates of *CDKN2A*, prone to co-occur with *KRAS* as well. Notably, *CDKN2A*, identified as a tumor-suppressor gene, plays an important role in the activation of *KRAS* to further promote the tumorigenesis and progression of PDAC, especially in cases with concurrent mutations in both *KRAS* and *CDKN2A* [45, 46]. Therefore, the higher mutation rates of *CDKN2A* and co-occurrent with *KRAS* may partially explain the unfavorable survival outcomes in PDAC patients with low TSR.

However, our study had some limitations. First, its retrospective nature has inherent limitations, even though we enrolled four large-scale cohorts to enhance the solidity of our conclusions. Second, we did not take detailed ACT regimen into consideration when investigating the prognostic value and role of the TSR in predicting response to ACT. Therefore, clinical data from prospective studies are required in future studies to validate these results.

TSR was observed to be correlated with clinical outcomes in patients with PDAC, owing to the interactions of TSR with molecular and immune-microenvironment alterations. The TSR was examined to evaluate the LNM status with satisfactory accuracy in both surgically resected and EUS-FNA biopsy specimens regardless of clinical intervention, enhanced when combining with PD-L1 TPS and serum CA19-9 levels. As a result of these findings, the TSR was high recommended to be standardized and integrated into prospective clinical trials to further validate its potential in prediction of LNM and therapy response.

## Supplementary information

Supplementary

## Data Availability

All data generated or analyzed during this study are included in this published article and its supplementary information files. The code used and/or analyzed during the current study are available from the corresponding author on reasonable request.
