# Supplementary material for: Tumor-stroma ratio combined with PD-L1 identifies pancreatic ductal adenocarcinoma patients at risk for lymph node metastases
Source: Br J Cancer. 2025 Apr 18;132(12):1131–40. doi: 10.1038/s41416-025-03019-z (PMC12152126; doi:10.1038/s41416-025-03019-z)

Supplemental Table S1. Association of TSR with clinicopathological characteristics in the training, validation, and EUS-FNA cohort

Variables	Training Cohort				Validation Cohort				EUS-FNA Cohort			
	**N**	**TSR-Low**	**TSR-High**	***P***	**N**	**TSR-Low**	**TSR-High**	***P***	**N**	**TSR-Low**	**TSR-High**	***P***
**Age**				0.811				0.442				0.123
0	42	21(50.0)	21(50.0)		120	75(62.5)	45(37.5)		34	21(61.8)	13(38.2)	
1	63	30(47.6)	33(52.4)		152	88(57.9)	64(42.1)		41	18(43.9)	23(56.1)	
**Sex**				0.946				0.086				0.912
0	47	23(48.9)	24(51.1)		125	68(54.4)	57(45.6)		37	19(51.4)	18(48.6)	
1	58	28(48.3)	30(51.7)		147	95(64.6)	52(35.4)		38	20(52.6)	18(47.4)	
**Differentiation**				0.214				0.252				
G1-G2	70	31(44.3)	39(55.7)		171	98(57.3)	73(42.7)			NA		
G3-G4	35	20(57.1)	15(42.9)		101	65(64.4)	36(35.6)					
**Location**				0.503				0.008				0.143
Head	55	25(45.5)	30(54.5)		166	110(66.3)	56(33.7)		48	28(58.3)	20(41.7)	
Body & Tail	50	26(52.0)	24(48.0)		106	53(50.0)	53(50.0)		27	11(40.7)	16(59.3)	
**CA199**				0.690				0.082				0.834
<37	29	15(51.7)	14(48.3)		58	29(50.0)	29(50.0)		20	10(50.0)	10(50.0)	
≥37	76	36(47.4)	40(52.6)		214	134(62.6)	80(37.4)		55	29(52.7)	26(47.3)	
**PNI**				0.214				0.095				0.617
Yes	70	37(52.9)	33(47.1)		183	116(63.4)	67(36.6)		48	26(54.2)	22(45.8)	
No	35	14(40.0)	21(60.0)		89	47(52.8)	42(47.2)		27	13(48.1)	14(51.9)	
**LVI**				0.042				0.213				0.608
Yes	43	26(60.5)	17(39.5)		102	66(64.7)	36(35.3)		29	14(48.3)	15(51.7)	
No	62	25(40.3)	37(59.7)		170	97(57.1)	73(42.9)		46	25(54.3)	21(45.7)	
**ACT**				0.692				0.506				0.091
Yes	78	37(47.4)	41(52.6)		191	112(58.6)	79(41.4)		49	22(44.9)	27(55.1)	
No	27	14(51.9)	13(48.1)		81	51(63.0)	30(37.0)		26	17(65.4)	9(34.6)	
**pT Stage**				0.208				0.331				0.729
T1-T2	74	33(44.6)	41(55.4)		201	117(58.2)	84(41.8)		57	29(50.9)	28(49.1)	
T3-T4	31	18(58.1)	13(41.9)		71	46(64.8)	25(35.2)		18	10(55.6)	8(44.4)	
**pN Stage**				**<0.001**				**<0.001**				**0.003**
N0	49	13(26.5)	36(73.5)		103	32(31.1)	71(68.9)		41	15(36.6)	26(63.4)	
N1-N2	56	38(67.9)	18(32.1)		169	131(77.5)	38(22.5)		34	24(70.6)	10(29.4)	
**pM Stage**				0.525				0.675				0.333
M0	102	49(48.0)	53(52.0)		263	157(59.7)	106(40.3)		74	38(51.4)	36(48.6)	
M1	3	2(66.7)	1(33.3)		9	6(66.7)	3(33.3)		1	1(100.0)	0(0.0)	
**AJCC**				**0.005**				**<0.001**				**0.001**
I-II	89	38(42.7)	51(57.3)		215	116(54.0)	99(46.0)		64	28(43.8)	36(56.3)	
III-IV	16	13(81.3)	3(18.8)		57	47(82.5)	10(17.5)		11	11(100.0)	0(0.0)	

AJCC, American Joint Committee on Cancer; PNI, perineural invasion; LVI, lymphovascular invasion; ACT, adjuvant chemotherapy; TSR, tumor-stroma ratio; EUS-FNA, Endoscopic ultrasound - guided fine needle aspiration; NA, not available.

p values < 0.05 are bolded.

Supplemental Table S2. Association of TSR with clinicopathological characteristics in the Pre-Neoadjuvant therapy EUS-FNA biopsy cohort

Variables	N	TSR Low	TSR High	*P*
**Age**				0.958
<60, yrs	38	15(39.5)	23(60.5)	
≥60, yrs	65	26(40.0)	39(60.0)	
**Sex**				**0.046**
Female	45	13(28.9)	32(71.1)	
Male	58	28(48.3)	30(51.7)	
**Location**				**0.025**
Head	59	29(49.2)	30(50.8)	
Body & Tail	44	12(27.3)	32(72.7)	
**Pre-Neoadjuvant therapy CA19-9**				0.763
<37	26	11(42.3)	15(57.7)	
≥37	77	30(39.0)	47(61.0)	
**pT Stage**				**<0.001**
T1-T2	40	7(17.5)	33(82.5)	
T3-T4	63	34(54.0)	29(46.0)	
**pN Stage**				**0.044**
N0	87	31(35.6)	56(64.4)	
N1	16	10(62.5)	6(37.5)	
**AJCC**				**<0.001**
I	28	0(0.0)	28(100.0)	
II	75	41(54.7)	34(45.3)	
**Neoadjuvant therapy**				0.529
Gemcitabine + Radiation	96	39(40.6)	57(59.4)	
Gemcitabine + S-1 + Radiation	7	2(28.6)	5(71.4)	
**Pathological response**				**<0.001**
Non-responder	58	34(58.6)	24(41.4)	
Responder	45	7(15.6)	38(84.4)	

AJCC, American Joint Committee on Cancer; EUS-FNA, Endoscopic ultrasound - guided fine needle aspiration; TSR, tumor-stroma ratio.
p values < 0.05 are bold.

Supplemental Table S3. Univariate Cox analysis of TSR and clinicopathological characteristics in the training, validation, and EUS-FNA cohort

Variables	Training		Validation		EUS-FNA	
	HR(95%CI)	*p*	HR(95%CI)	*p*	HR(95%CI)	*p*
**Age**						
<60, yrs	reference		reference		reference	
≥60, yrs	1.55 (0.93 - 2.58)	0.092	1.18 (0.87 - 1.60)	0.283	1.02 (0.57 - 1.83)	0.946
**Sex**						
Female	reference		reference		reference	
Male	1.11 (0.68 - 1.78)	0.683	1.36 (1.00 - 1.84)	**0.048**	1.03 (0.58 -1.82)	0.918
**Differentiation**						
moderate & well	reference		reference		NA	
Poor	2.17 (1.33 - 3.53)	**0.002**	2.01 (1.49 - 2.72)	**<0.001**	NA	
**Location**						
Head	reference		reference		reference	
Body & tail	0.91 (0.56 - 1.46)	0.687	0.64 (0.46 - 0.87)	**0.005**	0.62 (0.33 - 1.14)	0.122
**Preoperative CA19-9**						
<37	reference		reference		reference	
≥37	0.75 (0.45 - 1.27)	0.291	1.11 (0.77 - 1.60)	0.573	1.54 (0.79 - 3.02)	0.205
**PNI**						
No	reference		reference		NA	
Yes	1.58 (0.93 - 2.70)	0.094	1.14 (0.83 - 1.57)	0.420	NA	
**LVI**						
No	reference		reference		NA	
Yes	1.05 (0.65 - 1.71)	0.841	1.20 (0.89 - 1.63)	0.240	NA	
**ACT**						
No	reference		reference		reference	
Yes	0.53 (0.31 - 0.89)	**0.018**	0.52 (0.38 - 0.70)	**<0.001**	0.69 (0.38 - 1.25)	0.219
**pT Stage**						
T1-T2	reference		reference		reference	
T3-T4	1.79 (1.07 - 3.00)	**0.028**	1.76 (1.28 - 2.44)	**<0.001**	2.84 (1.49 - 5.42)	**0.002**
**pN stage**						
N0	reference		reference		reference	
N1-N2	1.49 (0.92 - 2.43)	0.106	1.75 (1.26 - 2.41)	**<0.001**	1.53 (0.85 - 2.74)	0.153
**AJCC**						
I-II	reference		reference		reference	
III-IV	3.13 (1.68 - 5.82)	**<0.001**	2.08 (1.49 - 2.92)	**<0.001**	3.17 (1.55 - 6.49)	**0.002**
**TSR**						
High	reference		reference		reference	
Low	3.97 (2.38 - 6.63)	**<0.001**	4.41 (3.10 - 6.25)	**<0.001**	4.49 (2.35 - 8.57)	**<0.001**

EUS-FNA, Endoscopic ultrasound - guided fine needle aspiration; AJCC, American Joint Committee on Cancer; PNI, perineural invasion; LVI, lymphovascular invasion; ACT, adjuvant chemotherapy; TSR, tumor-stroma ratio; HR, hazard ratio; CI, confidence interval; NA, not available.
p values < 0.05 are bolded.

Supplemental Table S4. Univariate Cox analysis of TSR and clinicopathological characteristics in the Pre-Neoadjuvant therapy EUS-FNA biopsy cohort

Variables	HR(95%CI)	*p*
**Age**		
<60, yrs	reference	
≥60, yrs	1.06 (0.62 - 1.82)	0.835
**Sex**		
Female	reference	
Male	1.22 (0.72 - 2.05)	0.465
**Location**		
Head	reference	
Body & tail	0.58 (0.34 - 1.00)	0.051
**Pre-Neoadjuvant therapy CA19-9**		
<37, U/mL	reference	
≥37, U/mL	1.30 (0.69 - 2.45)	0.424
**ypT Stage (post-Neoadjuvant therapy)**		
T1-T2	reference	
T3-T4	6.07 (3.04 - 12.12)	**<0.001**
**ypN Stage (post-Neoadjuvant therapy)**		
N0	Reference	
N1	1.38 (0.69 - 2.73)	0.361
**AJCC**		
I	Reference	
II	7.61 (3.43 - 16.90)	**<0.001**
**Pathological response**		
CAP0-2	reference	
CAP3	5.46 (2.94 - 10.14)	**<0.001**
**Neoadjuvant treatment**		
Gemcitabine + Radiation	reference	
Gemcitabine +S1+Radiation	0.35 (0.08 - 1.44)	0.146
**TSR**		
High	reference	
Low	6.81 (3.74 - 12.41)	**<0.001**

AJCC, American Joint Committee on Cancer; EUS-FNA, Endoscopic ultrasound - guided fine needle aspiration; TSR, tumor-stroma ratio; HR, hazard ratio; CI, confidence interval.

p values < 0.05 are bolded.

Supplemental Figure S1. Molecular Characteristics of Different TSR in Patients with PDAC. A and B, Top 20 significantly mutated genes in the samples of different TSR subgroups obtained from the TCGA cohort. C, Co-occurrence among the top 20 mutated genes in the TSR-low (C) and -high (D) subgroups of patients with PDAC from the TCGA dataset. *P*-values < .05 are marked in red. TSR, tumor-stroma ratio; PDAC, pancreatic ductal adenocarcinoma; and TCGA, The Cancer Genome Atlas.

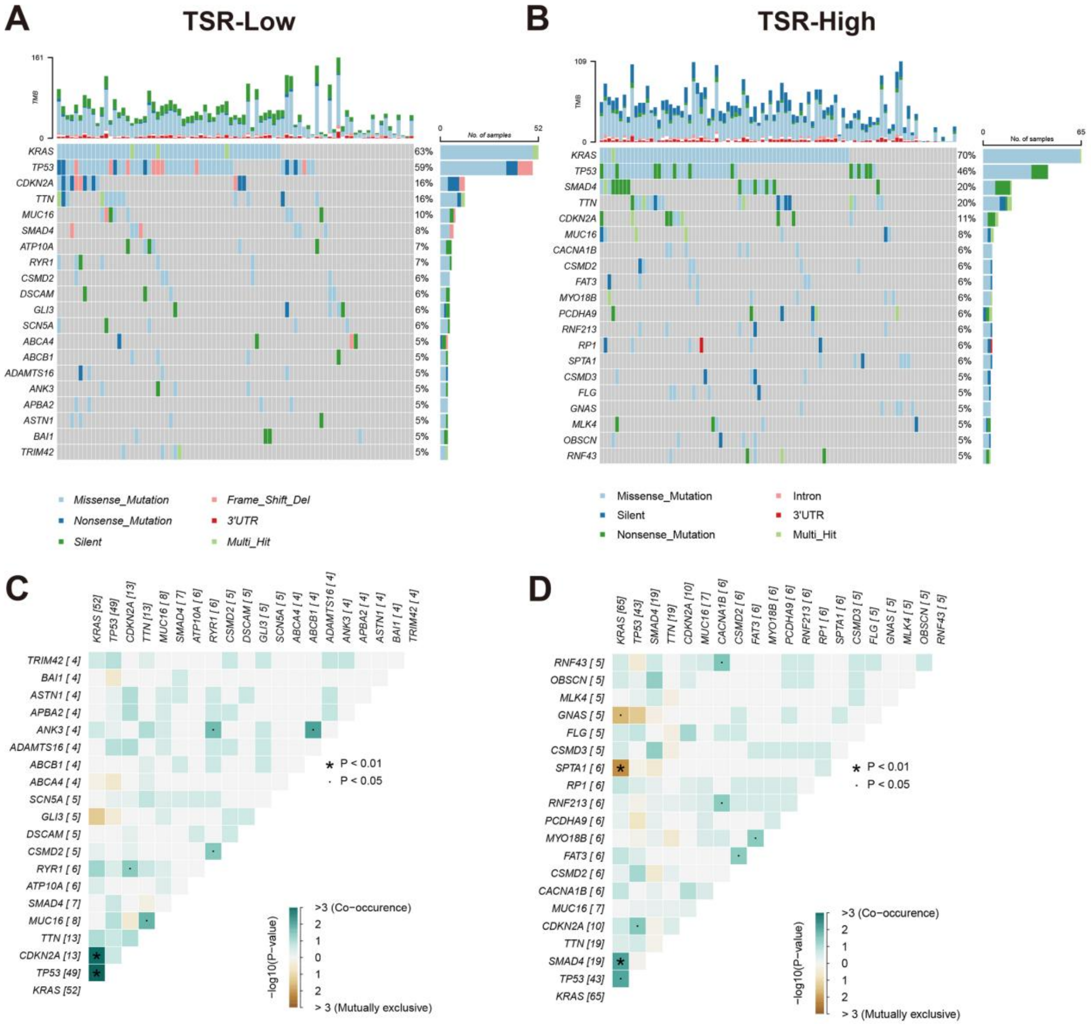

Supplement: Supplementary file 1 — Supplementary [file 41416_2025_3019_MOESM1_ESM.pdf]
